# Coffeeberry Activates the CaMKII/CREB/BDNF Pathway, Normalizes Autophagy and Apoptosis Signaling in Nonalcoholic Fatty Liver Rodent Model

**DOI:** 10.3390/nu13103652

**Published:** 2021-10-19

**Authors:** Meng-Chun Lu, I-Te Lee, Ling-Zong Hong, Eyal Ben-Arie, Yu-Hsuan Lin, Wei-Ting Lin, Pei-Yu Kao, Mei-Due Yang, Yin-Ching Chan

**Affiliations:** 1 Department of Clinical Nutrition, China Medical University Hospital, Taichung 406040, Taiwan; t10294@mail.cmuh.org.tw; 2 Department of Nutrition, China Medical University, Taichung 406040, Taiwan; 3 Department of Food and Nutrition, Providence University, Taichung 43301, Taiwan; g1050002@gm.pu.edu.tw (Y.-H.L.); pupuki520@gmail.com (W.-T.L.); 4 Division of Endocrinology and Metabolism, Taichung Veterans General Hospital, Taichung 40705, Taiwan; itlee@vghtc.gov.tw; 5 Department of Medical Research, Taichung Veterans General Hospital, Taichung 40705, Taiwan; lzhong1950@gmail.com; 6 Graduate Institute of Acupuncture Science, Collage of Chinese Medicine, China Medical University, Taichung 406040, Taiwan; benarie19@gmail.com; 7 Division of Thoracic Surgery, Department of Surgery, China Medical University Hospital, Taichung 406040, Taiwan; ludouto@gmail.com; 8 Division of General Surgery, Department of Surgery, China Medical University Hospital, Taichung 406040, Taiwan; d5218@mail.cmuh.org.tw

**Keywords:** nonalcoholic fatty liver disease, coffeeberry, redox status, CaMKII/CREB/BDNF, autophagy, apoptosis

## Abstract

Nonalcoholic fatty liver disease (NAFLD) shows extensive liver cell destruction with lipid accumulation, which is frequently accompanied by metabolic comorbidities and increases mortality. This study aimed to investigate the effects of coffeeberry (CB) on regulating the redox status, the CaMKII/CREB/BDNF pathway, autophagy, and apoptosis signaling by a NAFLD rodent model senescence-accelerated mice prone 8 (SAMP8). Three-month-old male SAMP8 mice were divided into a control group and three CB groups (50, 100, and 200 mg/kg BW), and fed for 12 weeks. The results show that CB reduced hepatic malondialdehyde and carbonyl protein levels. CB significantly enhanced Ca^2+^/calmodulin-dependent protein kinase II (CaMKII) and brain-derived neurotrophic factor (BDNF) and reduced the phospho-cAMP response element-binding protein (p-CREB)/CREB ratio. In addition, CB increased the silent information regulator T1 level, promoted Beclin 1 and microtubule-associated protein light chain 3 II expressions, and reduced phosphorylated mammalian target of rapamycin and its downstream p-p70s6k levels. CB also inhibited the expressions of apoptosis-related factors poly (ADP-ribose) polymerase-1 and the apoptosis-inducing factor. In conclusion, CB might protect the liver by reducing oxidative stress, activating the CaMKII/CREB/BDNF pathway, and improving autophagic and apoptotic expressions in a dose-dependent manner.

## 1. Introduction

With the modern western diet and the lack of exercise, nonalcoholic fatty liver disease (NAFLD) and its complications have increased worldwide, including in Asia and Taiwan. According to the Ministry of Health and Welfare report of Taiwan, chronic liver disease and liver cirrhosis were the tenth leading cause of death in 2020 [1]. Several studies on the general population and those undergoing a health checkup showed the prevalence of NAFLD ranging from 11.4% to 41% in Taiwan [2], while the estimated prevalence of NAFLD is 25.24% (95% CI: 22.10-28.65) worldwide [3]. Moreover, NAFLD is frequently accompanied by metabolic comorbidities, such as obesity and metabolic syndrome, and is a major cause of liver disease-related morbidity [3]. Therefore, the treatment and prevention of liver diseases, such as NAFLD, is an important issue that requires more attention.

NAFLD is mainly associated with the accumulation of fat in the liver, which causes cell lesions. Oxidative stress, lipid peroxidation, and cytokines play an important role in the NAFLD mechanism. Oxidative stress interacts with hepatic cells, resulting in degrees of hepatocyte damage, such as inflammatory responses, liver fibrosis, cirrhosis, and liver cell degeneration [4]. Recently, the CaMKII/CREB/BDNF pathway was noted to be a possibly modulatory factor associated with liver physiology. BDNF has been proven to improve fatty liver and pancreatic dysfunction in type 2 diabetic mice [5]. Chronic stress, such as inflammation, affects calcium/calmodulin-protein kinase II (CaMK II), CREB, and BDNF expression, and induces the dysfunction of calcium-ion regulation [6]. The inflammatory factors, such as iNOS, COX-2, TNF-α, and IL-1 β, are reduced by inhibiting p-CREB expression [7]. The CaMKII/CREB/BDNF pathway may play a role in lessening the inflammatory response and retarding the accumulation of fat in the liver.

Autophagy is a lysosomal degradative pathway that functions to promote cell survival by supplying energy in stress or removing damaged organelles and proteins after injury [8]. Autophagy contributes to liver homeostasis by controlling the quality of cytoplasm, as autophagy removes misfolded proteins and damaged organelles [9]. Moreover, autophagy also shows benefits in lipid metabolism through lipophagy [10]. Lipophagy is a lysosomal-autophagic pathway that plays an important part in the early steps of lipid degradation. Free fatty acids (FFAs) are generated by lipophagy, from the breakdown of triglycerides, and fuel cellular rates of mitochondrial *β*-oxidation [11]. Several signals are involved in modulating the autophagic function, such as the silent information regulator T1 (Sirt1), Beclin 1, and microtubule-associated protein light chain 3 II (LC3-II). Sirt1 is a nicotinamide adenine dinucleotide (NAD)-dependent class III histone deacetylase and is strongly linked to cell survival [12]. Sirt1 promotes the assembly of the autophagy-related genes (ATG) 5 and ATG12 in autophagy-related proteins by the Beclin 1 protein and increases the formation of auto-phagocytic activation index LC3-II [13,14]. In addition, Sirt1 also inhibits the mammalian target of rapamycin (mTOR) and its downstream P70S6K phosphorylation to enhance autophagy. mTOR is a serine/threonine-protein kinase that regulates cell metabolism, growth, proliferation, and survival [15]. Inhibited mTOR promotes the formation of the unc-51-like kinase (ULK)-1/2 complex and induces the progression of autophagy [16].

Apoptosis is another major component of programmed cell death (PCD), while an increasing number of studies support the existence of a caspase-independent pathway of PCD. Caspase-independent apoptosis is activated by oxidative stress or other factors. After activation, the poly (ADP-ribose) polymerase-1 (PARP-1) is secreted in the nucleus first, then translocates to the mitochondria and stimulates the releasing of the apoptosis-inducing factor (AIF) [17]. AIF will induce the condensation and fragmentation of DNA, which eventually leads to apoptosis [17].

Coffeeberry (CB) is an unprocessed coffee bean, and shows beneficial effects on anti-oxidation, collagen production, repairing aging skin, and increasing brain-derived neurotrophic factor (BDNF) levels [18,19,20,21]. Coffeeberry extract significantly enhanced blood antioxidant capacity and prevented exercise-induced oxidative stress during training periods [22]. Polyphenol-rich, non-caffeinated coffeeberry extract beverages were also reported to significantly attenuate the scores of self-reported fatigue and alertness [23]. The livers of senescence-accelerated mice prone 8 (SAMP8) mice are characterized by increased fatty degeneration, hepatocyte death, fibrosis, cirrhotic changes, inflammatory mononuclear cell infiltration, and sporadic neoplastic changes when compared with SAMR1 mice (normal control) [24]. SAMP8 mice also showed higher alanine aminotransferase (ALT) and aspartate aminotransferase (AST) than the SAMR1 mice [24]. Thus, the SAMP8 mouse strain is suggested to be a valuable animal model for the study of liver diseases [24]. In our previous study, we found CB enhanced the liver function by lowering the inflammatory signaling factors, including nuclear factor κB (NF-κB), inducible nitric oxide synthase (iNOS) and cyclooxygenase 2 (COX-2), reduced alanine aminotransferase (ALT) levels, and improved the histological changes by using SAMP8 mice [25]. However, the ability of CB to lessen the NAFLD process is still unclear. Thus, this study aimed to investigate the effects of CB on the related signaling of redox status, the CaMKII/CREB/BDNF pathway, autophagy, and apoptosis by using SAMP8 mice.

## 2. Material and Methods

### 2.1. Animals and Diets

SAMP8 (SAMP8/Ta Slc) mice were acquired from the Council for Senescence-Accelerated Mouse (SAM) Research, Japan, after confirming the genetic characteristics, and were maintained through inbreeding in the standard animal room at Providence University. Three-month-old SAMP8 mice were used and divided into a control group and three different CB groups using three different doses. The control group was fed with the American Institute of Nutrition (AIN) 93-M basal diet, while the CB groups were fed the American Institute of Nutrition (AIN) 93-M basal diet mixed with CB powder with doses of 50, 100, and 200 mg/kg BW/day, respectively, and were allowed free access to the experimental diets and drinking water for 12 weeks (*n* = 6/group). The mice were housed under controlled environmental conditions (22 ± 2 °C, 65% ± 5% relative humidity, 7:00–19:00 lighting period), as described in our previous study [26]. The food intake of the mice in each cage was recorded every day and the value was divided by the number of the mice in that cage to represent the mean food intake for one mouse. The weight of each mouse in each group was recorded every week until sacrifice. The study protocol was approved by the Animal Research Ethics Committee at Providence University, Taichung, Taiwan (20160607-A06 and 20170808-A0).

### 2.2. Redox Status Analysis

The liver tissue of the sacrificed mice was first separated; part of the tissue was instantly inserted into a sodium phosphate buffer (100 mM; pH 7.4), homogenized, and centrifuged at 3000× *g* in a refrigerated centrifuge (Hettich Universal 16 R, Tuttlingen, Germany) for 10 min. The assembled supernatants were assessed for lipid peroxide and protein oxidation in the exact method described by Chan & Hwang [26]. Lipid peroxidation levels were measured by blending the supernatants with 2′-thiobarbituric acid (4 g/kg in 0.2 M of HCl) and butylated hydroxytoluene (2 g/kg in 95% ethanol) at a ratio of 1:2:0.3. The blend was later heated at 90 °C for 45 min, left to cool down, and blended with 5 mL of n-butanol. The n-butanol layer was detached using centrifugation (1000× *g* for 10 min) and spectrophotometrically assayed for thiobarbituric acid-reactive substances (TBARS) at 532 nm. The results were demonstrated as µmol equivalents of malondialdehyde (MDA) per gram of liver tissue; an MDA from tetra methoxy propane was implemented for the control. The methods of evaluating carbonyl protein levels were also identical to the methods of the Chan & Hwang study [26]. Two 10 μL of tissue sample was then placed in duplicate in 1 mL of phosphate buffer (10 mM of Na_2_HPO_4_; pH 6.8). The two duplicates were then separated when one was treated with 0.2 mL of 2,4-Dinitorophenylhydrazine (10 mM in 2 N of HCl), and one was treated with 0.2 mL of 2 M HCl and used as a blank; as a result, both samples have their own blank sample. The blend was incubated for 1 h at room temperature and later followed by the addition of 1.2 mL of 20% trichloroacetic acid. The samples were cooled down in ice for 10 min and later centrifuged at 2800 rpm for 10 min; this allowed the supernatant to be discarded. The protein pellet was washed 2 times with 3 mL of ethyl alcohol–ethyl acetate blend (1:1, v/v). The solvent was then discarded, and nitrogen gas was used to dry the protein pellet. 1 mL of sodium phosphate (pH 6.8, 10 mM of Na_2_HPO_4_ containing 3% sodium dodecyl sulfate) was implemented to dissolve the protein pellet. The treated samples were compared against each sample blank in a scanner from 320 to 410 wavelengths in a spectrophotometer (Ultrospec 1100 Pro, Amersham Biosciences, Piscataway, NJ, USA). 360 nm (±10 nm) was appointed as the peak absorbance to calculate protein carbonyl. The carbonyl groups concentration was appraised using the absorbance of 1 nmol/mL of carbonyl at 360 = 0.022. The Pierce Micro BCA protein assay kit was used to conduct a protein assay assessment. The total amount of protein data was expressed as the nmol carbonyl protein formed per mg.

### 2.3. Western Blotting

Western blotting was performed as described in our previous study [27]. The total protein samples were extracted from liver tissues, and their protein concentrations were evaluated using the bicinchoninic acid (BCA) method (Bio-Rad, Hercules, CA, USA). Protein electrophoresed on 10% (*w*/*v*) SDS polyacrylamide gels were transferred onto polyvinylidene difluoride (PVDF) membranes and blocked with TBST (TBS with 0.1% Tween-20) containing a 5% nonfat dry milk or 5% bovine serum albumin (BSA) for 1 h. After blocking, membranes were kept overnight at 4 °C in a blocking solution containing different primary antibodies, including anti-CaMKⅡ(1:1500, Cell Signaling, Danvers, MA, U SA), anti-p-CREB(1:1000, Cell Signaling, Danvers, MA, USA), anti-CREB(1:1000, Cell Signaling, Danvers, MA, USA), anti-p-CREB(1:1000, Cell Signaling, Danvers, MA, USA), anti-BDNF(1:3000, Burlingame, CA, Abcam, USA), anti-Sirt 1 (1:2000, King of Prussia, PA, CSL, USA), anti-Beclin 1 (1:3000, Irvine, California, GeneTex, USA), anti-LC3B (1:3000, Burlingame, CA, Abcam, USA), anti-mTOR(phosphor S2448) (1:3000, Burlingame, CA, Abcam, USA), anti-p-P70S6K (1:1000, King of Prussia, PA, CSL, USA), anti-AIF (1:10000, King of Prussia, PA, CSL, USA), anti-PARP 1 (1:10,000, King of Prussia, PA, CSL, USA), and anti-β actin (1:50,000, King of Prussia, PA, CSL, USA). The membranes were exposed to secondary antibodies after being washed with TBST at room temperature for 1 h. Immunoreactivity was detected using enhanced chemiluminescence (ECL) reagents. Quantitative results were performed using a FusionCapt Advance Camera and a FusionCapt Advance Analyzer (version 16.07, Sursee, Switzerland).

### 2.4. Statistics

The SPSS software (SPSS Inc., Chicago, IL, USA) was used to analyze the results. The main statistical test used to assess the statistical difference was the one-way analysis of variance (ANOVA). The student’s t-test was used to compare the two groups in cases where the F-test was significant. Data are displayed as mean ± standard error of mean (SEM) and the results were classified as significant if the *p*-value was lower than 0.05 (*p* < 0.05).

## 3. Results

### 3.1. CB Reduced the Oxidative Status

**Figure 1:**
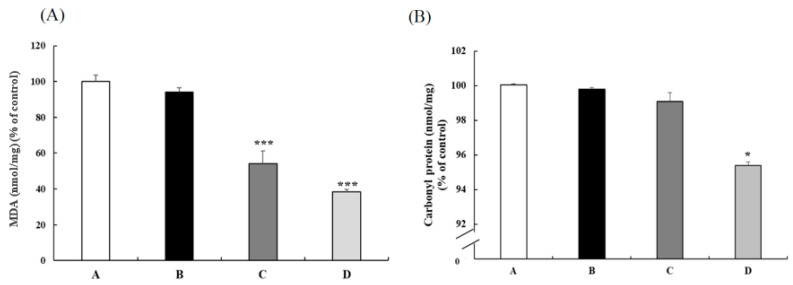
The (**A**) MDA and (**B**) carbonyl protein levels in the livers of 3-month-old male SAMP8 mice fed with different diets for 12 weeks. Values are displayed as mean ± S.E.M. and were analyzed by one-way ANOVA (*n* = 6/group). * *p* < 0.05, *** *p* < 0.001 compared to the control group. A = SAMP8 control, B= 50 mg/kg BW/day CB, C = 100 mg/kg BW/day CB, D = 200 mg/kg BW/day CB.

BW changes and food intakes did not differ among the four groups, while the related liver weight of three CB groups tended to be lower than the control but without a significant difference (Table 1). Compared with the SAMP8 control group, 100 and 200 mg/kg BW CB groups had significantly lower lipid peroxide (Figure 1A). Furthermore, the 200 mg/kg BW CB group had significantly decreased carbonyl protein levels (Figure 1B). These results reveal that CB could reduce oxidative stress in the NAFLD animal model.

### 3.2. CB Improved the CaMKII/CREB/BDNF Signaling

**Figure 2:**
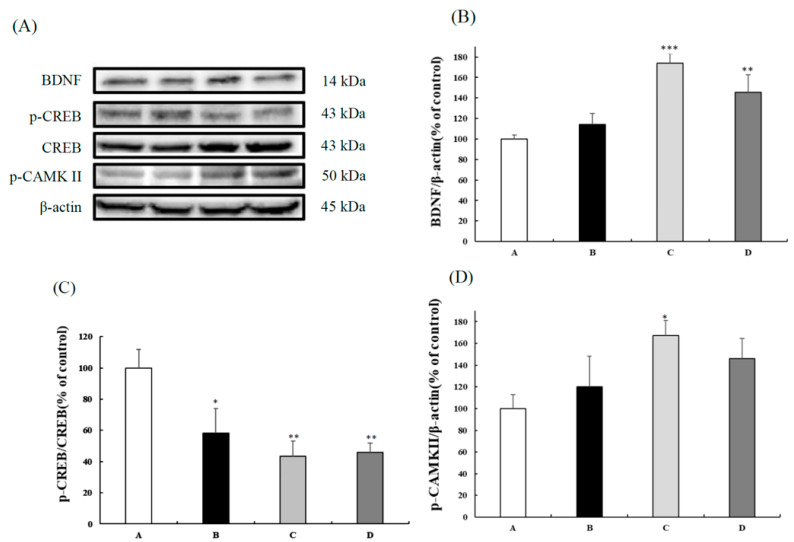
Protein expressions of the livers in 3-month-old male SAMP8 mice fed with different diets for 12 weeks. (**A**) The gel picture BDNF, p-CREB, total CREB, p-CAMK II, and β-actin expressions. (**B**) BDNF, (**C**) p-CREB/total CREB ratio, and (**D**) p-CAMK II expressions of the liver among different groups. The y axis quantified by the western blot analysis protein of BDNF/β-actin, p-CREB/CREB ratio, and p-CAMKII/β-actin are expressed as the percentage of control. Values are displayed as mean ± S.E.M. and were analyzed by one-way ANOVA (*n* = 6/group). * *p* < 0.05, ** *p* < 0.005, *** *p* < 0.001 compared to the control group. A = SAMP8 control, B = 50 mg/kg BW/day CB, C = 100 mg/kg BW/day CB, D = 200 mg/kg BW/day CB.

Three-month-old SAMP8 mice were administered CB (50 mg/kg BW, 100 mg/kg BW, 200 mg/kg BW). After 12 weeks of administration, liver lysates were immunoblotted with targeted antibodies. Compared to the control group, the 100 and 200 mg/kg BW CB groups had significantly increased BDNF expression and a lower p-CREB/CREB ratio (Figure 2B,C). The CAMKII levels of the three CB groups tended to be higher than those of the control group, while the significance was found only between the control and the 100 mg/kg BW group (Figure 2D). These data suggested that CB affected the expressions of CaMKII/CREB/BDNF signaling.

### 3.3. CB Enhanced the Autophagic Expressions

**Figure 3:**
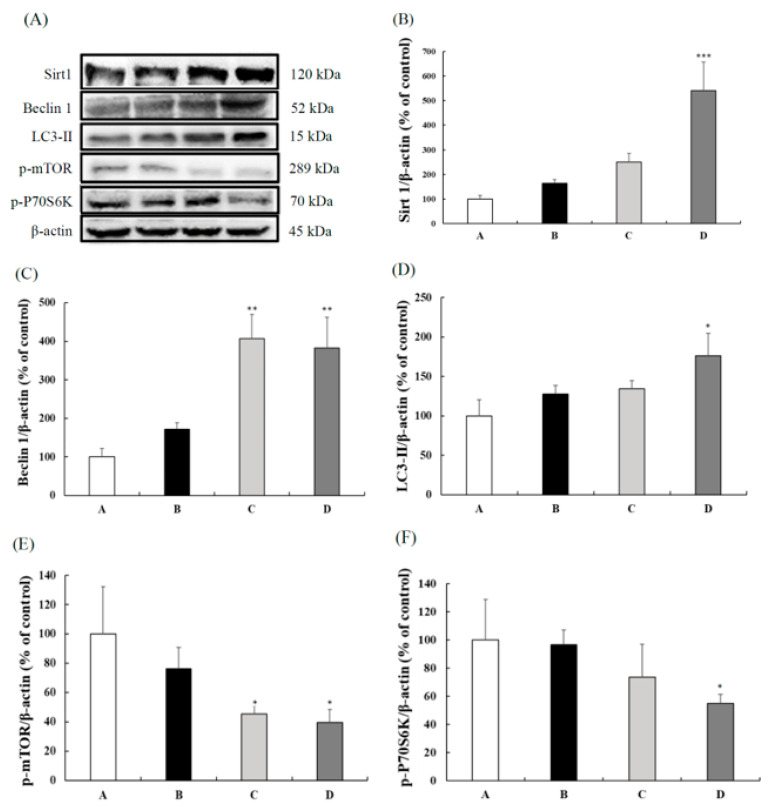
Protein expressions in the livers of 3-month-old male SAMP8 mice fed with different diets for 12 weeks. (**A**) The gel picture of Sirt 1, Beclin 1, LC3-II, p-mTOR, p-P70S6K, and β-actin expressions. (**B**) Sirt 1, (**C**) Beclin 1, (**D**) LC3-II, (**E**) p-mTOR, and (**F**) p-P70S6K protein expression in the livers of different groups. The y axis quantified by the western blot analysis protein of Sirt 1/β-actin, Beclin 1/β-actin, LC3-II/β-actin, p-mTOR/β-actin, and p-P70S6K/β-actin are expressed as the percentage of control. Values are displayed as mean ± S.E.M. and were analyzed by one-way ANOVA (*n* = 6/group). * *p* < 0.05, ** *p* < 0.005, *** *p* < 0.001 compared to the control group. A = SAMP8 control, B = 50 mg/kg BW/day CB, C = 100 mg/kg BW/day CB, D = 200 mg/kg BW/day CB.

Compared with the control group, the 200 mg/kg BW CB group had increased Sirt 1 expression (Figure 3B). The Beclin 1 and LC3-II protein expressions in the liver are shown in Figure 3C,D, respectively. Compared to the control group, 100 and 200 mg/kg BW CB supplementations significantly increased Beclin 1 expression, whereas only the 200 mg/kg BW CB group had significantly higher LC3-II expression than the control group. Figure 3E,F showed the p-mTOR and p-P70S6K protein expressions in the liver. The 100 and 200 mg/kg BW CB groups had significantly decreased p-mTOR expressions compared to the control group. In addition, the 200 mg/kg BW CB group had lower p-P70S6K expression than the other three groups. The above results indicate that CB increased the autophagy in a dose-dependent manner by enhancing Beclin 1 and LC3-II expressions via inhibiting the expressions of p-mTOR and p-P70S6K protein.

### 3.4. CB Inhibited the Caspase-Independent Apoptosis

**Figure 4:**
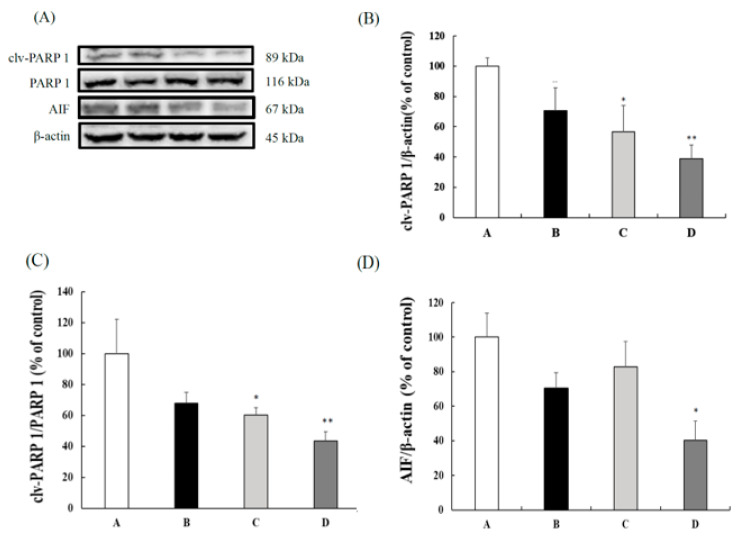
Protein expressions in the livers of 3-month-old male SAMP8 mice fed with different diets for 12 weeks. (**A**) The gel picture of cleaved-PARP 1, PARP 1, AIF, and β-actin expressions. (**B**) cleaved-PARP 1, (**C**) cleaved-PARP 1/PARP 1 ratio, and (**D**) AIF protein expression in livers of different groups. The y axis quantified by the western blot analysis protein of cleaved-PARP 1/β-actin, cleaved-PARP 1/PARP 1 ratio, and AIF/β-actin, expressed as the percentage of control. Values are displayed as mean ± S.E.M. and were analyzed by one-way ANOVA (*n* = 6/group). * *p* < 0.05, ** *p* < 0.005 compared to the control group. A = SAMP8 control, B = 50 mg/kg BW/day CB, C = 100 mg/kg BW/day CB, D = 200 mg/kg BW/day CB.

In this study, we also evaluated the effects of CB on the caspase-independent apoptotic factors. When compared with the SAMP8 control group, the 100 and 200 mg/kg BW CB groups showed a significantly lower cleaved-PARP 1 and cleaved-PARP 1/PARP 1 ratio (Figure 4B,C). Furthermore, the 200 mg/kg BW CB group had significantly reduced AIF expression when compared to the control group (Figure 4D). These data suggest that CB enhanced the apoptosis reaction by downregulating the PARP 1 and AIF expressions.

## 4. Discussion

Our previous study showed that CB reduced ALT level and improved the fatty liver score of SAMP8 mice by lowering the inflammatory signaling [25]. The present study further demonstrated that CB reduced the hepatic oxidative stress, enhanced CaMKII/CREB/BDNF and autophagic signaling, and inhibited the caspase-independent apoptosis. These results indicate CB as a potential agent for lessening the damage in liver disorder.

CaMKII/CREB/BDNF signaling is linked with cognitive and learning functions [28]. CaMKII phosphorylation activates signaling molecules and several other transcription factors including CREB [29], while CREB is associated with memory and synaptic plasticity that binds to the promoter regions of many genes [30]. Reduced intracellular p-CaMKII increases the intracellular CREB and p-CREB expression in the hippocampus, resulting in improved nuclear transcription activities of CREB [31]. CREB further alters the expression of BDNF, and BDNF is noted to play crucial roles in the nervous systems by promoting neurons differentiation, improving neurite outgrowth and synaptogenesis, and inhibiting apoptosis [32]. A study by Islam et al. [28] demonstrated that the oral administration of Theobromine, a primary methylxanthine found in cacao beans, improved the working memory by the upregulation of p-CaMKII, p-CREB, and BDNF levels. This indicates the importance of CaMKII/CREB/BDNF signaling in neuroprotection. However, the implicate of CaMKII/CREB/BDNF signaling on liver tissue, especially in liver disorder, is not clear yet.

A data-mining analysis revealed that BDNF is an important marker for the prevention and treatment of NAFLD [33]. BDNF level is significantly lower in patients with liver cirrhosis induced by hepatitis B virus (HBV) [34]. In the high-fat diet- (HFD) fed-mice, hepatic steatosis is induced by reducing the BDNF-TrkB expression [35]. Genzer et al. [36] investigated the comprehensive metabolic analysis of BDNF signaling in hepatocytes. The study demonstrated that BDNF treatment decreased CREB and GS3K proteins to inhibit gluconeogenesis and activate glycogen synthesis. In hepatocytes, CREB is a downstream target of BDNF, which is an endonuclear regulatory factor, regulating transcription by self-phosphorylation. CREB plays an important role in gluconeogenesis and fatty acid oxidation [37] and has been proven to reduce hepatic TG synthesis and storage during fasting via PPAR-γ repression [38]. The phosphorylation of CREB increases CAMK-II expression and then inhibits the proliferation of activated hepatic stellate cells [39]. Inhibited CaMKII expression reduces the phosphorylation of FoxO1, which leads the impairments of hepatic glucose homeostasis [40]. Our results found that the CB groups have significantly increased BDNF and CaMKII levels and reduced CREB expression. This indicates that the CaMKII/CREB/BDNF pathway is an important factor to modulate the physiological status of the liver.

On the other hand, autophagy is also reported to ameliorate the pathogenesis of NAFLD by lowering the hepatic lipid load by lipophagy [41]. In the NAFLD rat model, autophagy-related proteins LC3 and Beclin1 are significantly decreased at both the mRNA and protein levels [42]. HFD-induced obese mice also show reduced hepatic autophagic function reflected in reduced Beclin-1 and LC3-II levels and decreased numbers of autophagosomes/autolysosomes [43]. Nonalcoholic steatohepatitis (NASH) patients have higher LC3-II, while the LC3-II level positively associates with the severity of liver disease. In addition, Sirt1 is proven to promote the expression of Beclin 1 and lead to the increase in auto-phagocytic activation index LC3-II [13,14]. Resveratrol improves hepatic steatosis by mediating the Sirt1/activating transcription factor 6 dependent mechanism [44]. Calorie restriction improves the NAFLD by enhancing the Sirt1 expression [45]. The above evidence indicates that autophagy plays a protective role during NAFLD. In this study, our results show that the supplementation of CB-enhanced Sirt1 expression promoted the formation of autophagosomes transformed by enhancing Belin 1 expression and increased the performance of autophagy marker protein LC3-II. Furthermore, Sirt1 also enhanced autophagy by inhibiting the phosphorylation of mTOR and its downstream P70S6K.

mTOR plays a key role in the regulation of lysosomal and autophagic acidification through the modulation of V-ATPase expression, and its use as an indicator to evaluate autophagic dysfunction in NAFLD has been suggested [46]. Autophagy occurs via the activation of mTOR signaling, contributing to its protective effects on liver ischemia/reperfusion injury [47]. Phytochemical may also achieve beneficial effects on hepatic protection by modulating the autophagic reaction. Coffee consumption is reported to decrease hepatic p-mTOR levels in aged mice [48]. Many studies have investigated the possible molecular mechanisms regarding coffee’s role in liver protection, most of them indicating the importance of caffeine and chlorogenic acid on lessening liver fibrosis [49]. Furthermore, caffeine and chlorogenic acid are also the active components in whole coffee fruit extract [50]. Caffeine is noted to enhance autophagic reaction via reducing PI3K/Akt/mTOR/p70S6K signaling [51]. Caffeine also increases the autophagy flux in hepatic stellate cells through the IRE1-α signaling pathway and inhibits the activation of hepatic astrocyte by blocking adenosine receptors in the experimental fibrosis model [49]. Chlorogenic acid is reported to enhance the sensitivity of human hepatocellular carcinoma cells lines to regorafenib treatment by inhibiting PI3K/Akt/mTOR signaling [52] and lessening liver injury by inhibiting autophagy in a rat model of NAFLD [53]. In this study, the supplementation of CB enhanced Sirt1 expression, promoted the formation of autophagosomes transformed by Belin 1 protein expression, and increased the performance of autophagy marker protein LC3-II. Increased Sirt1 expression also downregulated the phosphorylation of p-mTOR and its downstream P70S6K. This evidence indicates the beneficial effects of CB in enhancing the autophagic reactions in the livers of SAMP8 mice.

PARP-1, AIF, and endonuclease G (EndoG) play key roles in the caspase-independent apoptosis signaling pathway. Oxidative stress stimulates the nucleus to secrete PARP-1 and translocate to the mitochondria and promotes the AIF or Endo G on the mitochondria membrane to be released into the cytoplasm and nucleus, causing DNA condensation and fragmentation, leading to apoptosis [17]. PARP1 is activated in the non-alcoholic fatty liver of mice and patients. Inhibition of PARP1 activation reduces lipid accumulation and the inflammation of fatty liver in mice [54]. PARP inhibitors are noted to reduce hepatic triglyceride accumulation, metabolic disorders, inflammation, and fibrosis in preclinical models of liver disease [55]. PARP inhibitors also attenuate PARP activation and retard the development of liver damage in hepatitis models, and these benefits are related to Sirt1 [56]. Caffeine is noted to lower the survival of hepatic stellate cells (HSCs) by inducing apoptosis reaction [57]. Caffeine combined with 5-FU significantly increases the apoptotic level by up-regulating cleaved PARP and down-regulating Bcl-2 and Bcl-xL expressions in hepatocellular carcinoma (HCC) cells [58]. Caffeine induces glioma cell death, possibly via elevating the cleaved PARP-1/PARP-1 ratio and AIF expression [59]. Chlorogenic acid protects primary rat hepatocytes against palmitic acid-induced damage by reducing ER stress-mediated apoptosis [60] and prevents liver fibrosis and hepatoma by reducing oxidative pressure and modulating the homeostasis of glucose and lipids [49]. Our results demonstrate that CB supplementation increased Sirt 1 expression in a dose-dependent manner and reduced the levels of cleaved-PARP 1, cleaved-PARP 1/PARP 1 ratio, and AIF in the liver. These results show that upregulated Sirt1 expression may inhibit apoptosis by reducing PARP 1 and AIF releases.

Coffeeberry is derived from the whole fruit of the coffee plant, which contains valuable ingredients and has the potential to enhance nutrition and health, including antioxidant capacity, immune regulation, and tumor suppression [61]. CB has higher free-radical scavenging activity when compared with vitamin C or vitamin E [62]. Whole coffee fruit extract is proven to have great antioxidant properties with its high polyphenols, such as caffeine, chlorogenic acid, condensed proanthocyanidins, quinic acid, and ferulic acid [50]. The anti-obesity influence of coffee fruit extract may be due to its anti-adipogenic and lipolytic properties in 3T3-L1 adipocytes [63]. Coffee supplementation significantly reduces glutathione disulfide and MDA levels in the HFD diet group [64]. Caffeine, one component of CB, is consecutively metabolized in the liver into potentially active compounds by cytochrome P450 isoform CYP1A2, N-acetyl-transferase type 2, and xanthine oxidase [65], and has beneficial effects on inhibiting lipid peroxidation [66] and lowering the glutamyltransferase levels [67]. In addition, caffeine stimulates hepatic fat oxidation and shows potential lipolysis advantages in the NAFLD model through autophagy and significantly reduces hepatosteatosis through increasing liver β-oxidation, lipid uptake, and enhancing LC3-II and autophagic flux levels [68]. Our previous study demonstrated that CB lowered the ALT level, decreased the iNOS and COX-2 expressions, and improved fatty liver scores in SAMP8 mice [25]. In this study, we further found that CB lowered hepatic lipid peroxide and carbonyl protein levels, enhanced the CaMKII/CREB/BDNF and autophagic signaling, and reduced the apoptosis in a dose-dependent manner. However, the effective ingredients of CB in lessening liver disorder need further study.

We are the first to investigate the possible mechanism of CB by using a NAFLD rodent model. Our results reveal that CB supplementation reduced oxidative damage in the livers of SAMP8 mice, possibly through improving the CaMKII/CREB/BDNF pathway and autophagic signaling and downregulating the apoptosis factors. This evidence supports the potential benefits of CB in lessening the pathological progress of NAFLD.

## Figures and Tables

**Table 1:** Body weight gains, food intakes, and relative liver weight of 3-month-old male SAMP8 mice fed with different diet for 12 weeks.

Group	N	Weight Gain (gm)	Food Intakes (gm/day)	Liver (g/100g Body Weight)
A	6	3.08 ± 0.43	7.83 ± 0.16	4.36 ± 0.15
B	6	3.41 ± 0.32	7.51 ± 0.17	4.21 ± 0.20
C	6	3.42 ± 0.35	7.73 ± 0.49	4.20 ± 0.19
D	6	3.33 ± 0.38	7.69 ± 0.28	4.25 ± 0.13

Values are displayed as mean ± S.E.M. and were analyzed by one-way ANOVA. A = SAMP8 control, B = 50 mg/kg BW/day CB, C = 100 mg/kg BW/day CB, D = 200 mg/kg BW/day CB.

## Data Availability

Not applicable.
